# Steady-state responses to concurrent melodies: source distribution, top-down, and bottom-up attention

**DOI:** 10.1093/cercor/bhac260

**Published:** 2022-07-21

**Authors:** Cassia Low Manting, Balazs Gulyas, Fredrik Ullén, Daniel Lundqvist

**Affiliations:** Department of Clinical Neuroscience, Karolinska Institutet, Stockholm 17177, Sweden; Cognitive Neuroimaging Centre (CoNiC), Lee Kong Chien School of Medicine, Nanyang Technological University, Singapore 636921, Singapore; Department of Clinical Neuroscience, Karolinska Institutet, Stockholm 17177, Sweden; Cognitive Neuroimaging Centre (CoNiC), Lee Kong Chien School of Medicine, Nanyang Technological University, Singapore 636921, Singapore; Department of Neuroscience, Karolinska Institutet, Stockholm 17177, Sweden; Department of Cognitive Neuropsychology, Max Planck Institute for Empirical Aesthetics, Frankfurt 60322, Germany; Department of Clinical Neuroscience, Karolinska Institutet, Stockholm 17177, Sweden

**Keywords:** ASSR, cocktail party, MEG, music, simultaneous

## Abstract

Humans can direct attentional resources to a single sound occurring simultaneously among others to extract the most behaviourally relevant information present. To investigate this cognitive phenomenon in a precise manner, we used frequency-tagging to separate neural auditory steady-state responses (ASSRs) that can be traced back to each auditory stimulus, from the neural mix elicited by multiple simultaneous sounds. Using a mixture of 2 frequency-tagged melody streams, we instructed participants to selectively attend to one stream or the other while following the development of the pitch contour. Bottom-up attention towards either stream was also manipulated with salient changes in pitch. Distributed source analyses of magnetoencephalography measurements showed that the effect of ASSR enhancement from top-down driven attention was strongest at the left frontal cortex, while that of bottom-up driven attention was dominant at the right temporal cortex. Furthermore, the degree of ASSR suppression from simultaneous stimuli varied across cortical lobes and hemisphere. The ASSR source distribution changes from temporal-dominance during single-stream perception, to proportionally more activity in the frontal and centro-parietal cortical regions when listening to simultaneous streams. These findings are a step forward to studying cognition in more complex and naturalistic soundscapes using frequency-tagging.

## Introduction

The brain’s limited capacity constrains it from processing all incoming sensory information at the same time, employing attentional mechanisms to filter and select only the most relevant stimuli amidst a complex and dynamic environment. In the auditory domain, this is a cognitively demanding task that involves identifying disparate sound sources and directing attention exclusively to one or more selected sources, while suppressing interference from other ongoing sources competing for attention. The most classic example used to describe this phenomenon is the “cocktail party effect (CPE)” (Cherry 1953), wherein a listener makes a top-down driven shift of selective attention to the interlocutor, while concurrently suppressing bottom-up calls for attention from perceptually salient background noises, such as the loud clinks of glasses, bursts of laughter, and other ongoing conversations. Although the implications of selective attention on perception and performance are well-documented by a library of behavioral studies (Broadbent 1958; Treisman 1964), the neurobiological underpinnings of these effects are poorly understood and require further investigation using modern neuroimaging methodologies.

In magnetoencephalography (MEG) and electroencephalography (EEG), several techniques have been utilized to explore how the brain selectively attends and accomplishes the CPE. Most of these studies examined brain responses, known as event-related fields (ERFs), that are time-locked to the stimulus (Hansen et al. 1983; Woods et al. 1984; Wolff and Schröger 2001; Näätänen et al. 2007), although other studies used stimulus reconstruction (Mesgarani and Chang 2012; O'Sullivan et al. 2014) and auditory steady-state responses (ASSRs) (Bidet-Caulet et al. 2007; Bharadwaj et al. 2014). In the above studies, the effect of attention generally manifested as an enhanced amplitude or power of neural activity, more extensive neural interactions, or increased fidelity of neural encoding of the attended stimulus. Furthermore, for many of these studies, the analysis of attentional modulation centers around the primary auditory cortex, in particular the Heschl’s gyrus. However, a crucial and challenging problem in this field stems from the complexity of the neural activity mix that is elicited when multiple auditory stimuli are simultaneously presented—a scenario that is incompatible with the common ERF approach due to the difficulty of separating simultaneous ERFs originating from different sources. The separation of the resultant mixed neural signals to their constituent sources is necessary to allow researchers to study each individual stimulus-specific brain activity, such as activity generated by a particular instrument within an orchestra or a single voice in a choir or noisy CPE setting. Despite being a difficult step, the development of effective means to separate and identify mixed neural activities is crucial for the advancement of research in human cognitive abilities towards more complex scenarios containing simultaneous sounds.

A potential solution to this challenge involves the use of frequency-tagging to generate brain responses that are specific to each constituent auditory stimulus within a multistimuli mixture. This method makes use of the time- and phase-locking of neural activity to that of the driving stimulus, producing an oscillatory neural response known as the ASSR (Regan 1989; Ross et al. 2000; Picton et al. 2003). By assigning a unique driving frequency to each stimulus—commonly via adjusting the stimulus’ presentation rate or amplitude envelope as in amplitude-modulation (AM) frequency-tagging—the individual ASSR elicited by each stimulus can be resolved precisely from the recorded brain data using power spectral density (PSD) estimation techniques such as Fourier analysis (Lins and Picton 1995; Picton et al. 2003). Ideally, this method provides scientists that are interested in auditory research with a simple, efficient, and clean method of extracting neural responses to individual stimuli within a complex auditory mix. With careful design, this method can be used with a large range of concurrent natural auditory stimuli, such as instrument tones and human voices, potentially steering research away from typical simplistic and artificial experimental stimuli (e.g. clicks and beeps) to more naturalistic sounds that more closely resemble our real-life soundscape.

In the arena of auditory selective attention, frequency-tagging has shown considerable success in extracting the modulating effect of attention. For instance, the ASSR has been shown to increase with selective attention in paradigms that direct participants’ attention to a single sound within a mixture (Müller et al. 2009; Lazzouni et al. 2010; Bharadwaj et al. 2014;Manting et al. 2020 ; Manting et al. 2021). At source level, most studies (Bidet-Caulet et al. 2007; Müller et al. 2009; Lazzouni et al. 2010; Bharadwaj et al. 2014) have shown that the attentional enhancement of ASSRs is manifested in the auditory cortices. More recently however, our group has demonstrated that the neural activity enhancement from selective attention occurs also in regions beyond the auditory cortex, such as the parietal and frontal cortices, with regions in the prefrontal cortex experiencing the largest degree of ASSR enhancement (Manting et al. 2020, 2021). In addition, we also discovered that both the ASSR power itself and its degree of attentional modulation correlate with subjects’ musicality (Manting et al. 2021). Together, these findings reinforce the “gain” theory of selective attention (Hillyard et al. 1973, 1998; Mesgarani and Chang 2012; Ding and Simon 2012a, 2012b), wherein attention enhances the neural representation of a stimulus, and also show that selective attention recruits processes widespread across neural regions, some of which are influenced by long-term training. These results suggest that ASSRs can be utilized to better characterize and understand the development of cognitive abilities such as sound separation and selective attention, as well as to devise more efficient methods for the acquisition or enhancement of these skills.

Even though previous work on ASSRs has brought insights into the study of human cognition, one may argue that the paradigms used were too simplistic and thus not representative enough of the complexity of the cocktail party problem in reality, as our natural auditory environment often contains several simultaneous sounds and is more stimulating and chaotic. For example, most studies that have examined attentional enhancement on the ASSR used only a single auditory stimulus (Ross et al. 2004; Saupe et al. 2009), or 2 different auditory stimuli presented dichotically (Müller et al. 2009; Lazzouni et al. 2010; Mahajan et al. 2014). When the ASSR was applied to more complex auditory settings, the results were inconsistent (Gander et al. 2010; Riecke et al. 2014). This may not come as a surprise as the behavior of the ASSR seems to vary across different experimental parameters and paradigms. For instance, the ASSR waveform changes with type of stimulus (Picton et al. 2003), and while several ASSRs can be recorded simultaneously and clearly separated in frequency space, reductions in ASSR power levels were observed as the number of stimuli increased (John et al. 1998), demonstrating an inhibitory effect on power levels from simultaneous auditory stimuli. Such changes directly influence signal-to-noise ratios for ASSRs and increase the minimum recording time needed to identify and separate individual ASSRs, possibly making it problematic or even infeasible to directly apply the use of the ASSR from one experimental setting to another, such as in the case of comparing single-stimulus to multistimuli designs. Hence, it is important to study how the stimulus complexity influences the manifestation of the ASSR, in order to adapt the use of ASSRs in different situations and study designs. For example, despite the inhibitory effect of simultaneous ASSRs, with proper experimental adjustments, simultaneous ASSRs have nevertheless been proven useful for significantly speeding up hearing tests (Lins et al. 1996; Luts and Wouters 2004; Chou et al. 2012). Such adjustments may however be further complicated by the fact that ASSR is generated by a collection of cortical sources across the frontal, parietal and temporal lobes, which differ in strength as well as how they are modulated by behavioral and individual factors like selective attention and musicality, respectively. Furthermore, it is unclear how the suppression from simultaneous sounds influences these ASSR sources and their modulation by attention. Indeed, while the effect of simultaneous suppression per se is already well-established in present literature (Lins and Picton 1995; John et al. 1998), it has only been demonstrated at an overall sensor level but not region-specific across the cortical source space. Since both the ASSR power and ASSR modulation are unevenly distributed across the cortex, it is reasonable to expect that the effect of simultaneous suppression is also not homogenous across these sources. To extend the usefulness of ASSRs to simultaneous sounds, it is important to investigate how the suppression effect from simultaneous ASSRs affects ASSR activity, its cortical source distribution, the separability of mixed ASSR signals), as well as the ASSR’s ability to assess attentional modulation. Potentially, a deeper understanding of multiple ASSRs could pave the way towards applying frequency-tagging to more complex and naturalistic soundscapes for translating experimental findings into real-life applications.

In this study, we aimed to characterize the differences in the ~40 Hz cortical ASSR generated by simultaneously overlapping versus nonoverlapping stimuli. To this end, we compared 3 main aspects of the ASSR elicited by overlapping and nonoverlapping melody streams, namely the (i) power, (ii) source distribution, and (iii) modulation effects of selective attention, driven by both top-down and bottom-up factors. Participants were engaged in a selective attention task where they were instructed to exclusively focus on a single melody stream in a mixture of 2 simultaneously overlapping AM frequency-tagged streams that were presented diotically (i.e. identically to both ears), as we recorded the neural activity with MEG. We compared this dataset with a prior dataset using the same task instructions but with melody streams that were completely nonoverlapping in time. We hypothesize that a global reduction in ASSR power will be observed in the overlapping case compared to the nonoverlapping case, in lieu with earlier work that observed lower ASSR power in simultaneous sounds (Picton et al. 1987; Lins et al. 1996; John et al. 1998). We also hypothesize that the observed ASSR in the overlapping case will be modulated to a lesser degree by top-down selective attention due to decreased sensitivity with lower signal-to-noise ratios, and that the attentional enhancement will be most apparent in the frontal cortical regions, since our previous studies using nonoverlapping sources showed that the largest increase in ASSR power due to top-down selective attention was at the frontal regions (Manting et al. 2020). As for bottom-up driven attention, we hypothesize that the enhancement will be manifested primarily at the temporal cortices where automatic lower-level auditory processing typically takes place.

## Materials and methods

This paper involves the analysis of 2 different datasets—the present dataset acquired during the overlapping paradigm that is described under this section, and a separate dataset acquired using nonoverlapping melody streams. The latter dataset has previously been used in an earlier publication (Manting et al. 2021), albeit for a different research question.

### Experimental tasks: overlapping melody development tracking task

**Fig. 1:**
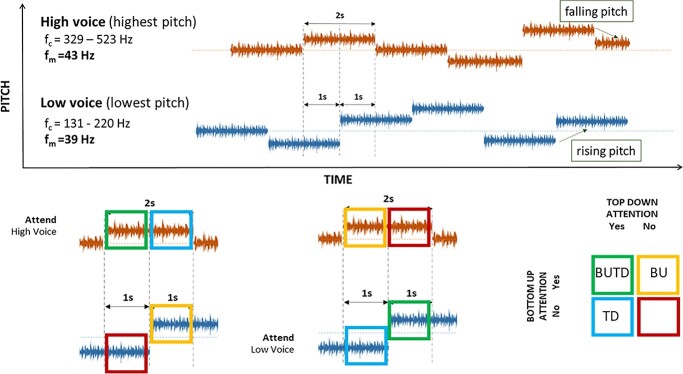
(Top row) Sample block of melody in the overlapping MDT task. Participants listened to 2 overlapping melody streams while attending to either the Low voice or High voice following a cue. When the melody stopped, participants reported the last direction of pitch change for the attended melody stream (i.e. falling, rising or constant pitch). The respective *f*_c_ (pitch) range and *f*_m_ of each stream are indicated above. (Bottom row) Color matrix describing how top-down (TD) and bottom-up (BU) attention was studied using the overlapping MDT paradigm. For the top-down driven attended voice, the tone onset (pitch change) draws bottom-up selective attention towards it in the first 1 s (green). At the second half of that attended tone, only top-down attention is still directed to the same voice (blue), while the simultaneous tone onset in the unattended voice generates a bottom-up pull on selective attention (yellow) in conflict with top-down attention. In the second half of the tone in the unattended voice, neither top-down nor bottom-up factors draw attention to the tone (red).

Using a modified version of the melody development tracking (MDT) task from our previous studies (Manting et al. 2020, 2021), participants were presented with 2 melody streams of different pitch (i.e. *f*_c_ range), namely the Low voice and High voice. In order to focus on feature-based sound separation (in this case, pitch and timing) rather than location-based (i.e. left-versus-right) source separation, the mixture of streams was presented identically to both ears via foam inserts. These melody streams were composed of a series of 2 s long tones and the onset of each voice occurs alternately (see Fig. 1), beginning either with the Low or with the High voice (order balanced across trials). Before each block of melody begins, the participants were cued to direct attention exclusively to the Low voice or High voice. When the melody stopped at a random time point, participants were required to report the last direction of pitch change for the attended voice (either falling, rising or constant pitch) with a button press. In total, 28 of these behavioral responses were collected over approximately 10 min of MEG recording time for each participant. During this time, 256 tone onsets were presented in each voice, with 128 onsets presented in the attend and unattend condition, respectively. Compared to the previous nonoverlapping experimental task (Manting et al. 2021), the current overlapping MDT task differs in that it contained 2 instead of 3 voices and the voices overlap in time. In the nonoverlapping experiment, the 3 voices were completely separated in time and presented in the order of increasing or decreasing pitch. There are also no silent gaps between tones in the current experiment compared to the 250 ms interstimulus interval between 750 ms long tones previously.

With this paradigm, the effect of both top-down and bottom-up driven attention can be investigated. At the tone onset (pitch change) of a cued attended voice, both top-down and bottom-up attention factors draws selective attention to the onset voice (green). At the second half of that attended tone, only top-down attention is still directed to the same voice (blue), while the simultaneous tone onset in the unattended voice generates a bottom-up pull on selective attention (yellow) in conflict with top-down attention. In the second half of the tone in the unattended voice, neither top-down nor bottom-up factors draw attention to the tone (red). The largest attentional effect is expected when both top-down and bottom-up factors align and draw attention towards the same direction.

### Stimuli

Each of the 2 voices was constructed using a stream of 2 s long sinusoidal tones of f_c_ between 131 and 523 Hz (Low voice: 131–220 Hz; High voice 329–523 Hz), generated using the Ableton Live 9 software (Berlin, Germany). These tones were amplitude-modulated sinusoidally in Ableton Live 9 at *f*_m_ of 39 (Low voice) and 43 (High voice) Hz, and a modulation depth of 100% to achieve maximum ASSR power (Ross et al. 2000). Sinusoidal amplitude modulation of the tone is carried out by modifying its amplitude envelope, which corresponds to its raw sound volume, with regular increases and decreases according to a sine wave. For simplicity, only tones in the C major harmonic scale were used. The duration of melody presentation was randomized to be between 10 and 28 s long to reduce predictability of the stop point and thereby maintain high attention throughout the melody. The volume (i.e. loudness) of the High voice was reduced by 10 dB relative to the Low voice to account for differences in subjective loudness for different frequency ranges (Robinson and Dadson 1956). The stimulus was presented identically via ear tubes to both ears with the volume calibrated to approximately 75 dB SPL per ear using a sound meter (Type 2235, Brüel & Kjær, Nærum, Denmark), subjected to individual comfort level. Apart from tone duration (2 s overlapping tones versus 750 ms nonoverlapping tones), the aforementioned parameters were identical for both the current overlapping and previous nonoverlapping experiments.

### Participants

A total of 28 participants with normal hearing volunteered to take part in the experiment (age 18–49 years, mean age = 28.6, SD = 6.2; 9 female; 2 left-handed). Two participants were excluded due to below-chance performance in the behavioral task, resulting in 26 participants for all sensor-space MEG analyses. For source-space MEG analyses, a third participant was further excluded due to unsuccessful MRI collection, resulting in a final sample of 25 participants. The experiment was approved by the Regional Ethics Review Board in Stockholm (Dnr: 2017/998-31/2). Both written and oral informed consent were obtained from all participants prior to the experiment. All participants received a monetary compensation of SEK 600 (~EUR 60). These participants also took part in the previously published nonoverlapping experiment (Manting et al. 2021).

### Data acquisition

MEG measurements were carried out using a 306-channel whole-scalp neuromagnetometer system (Elekta TRIUXTM, Elekta Neuromag Oy, Helsinki, Finland). Data were recorded at a 1 kHz sampling rate, on-line bandpass filtered between 0.1 and 330 Hz and stored for off-line analysis. Horizontal eye-movements and eye-blinks were monitored using horizontal and vertical bipolar electrooculography electrodes. Cardiac activity was monitored with bipolar electrocardiography electrodes attached below the left and right clavicle. Internal active shielding was active during MEG recordings to suppress electromagnetic artifacts from the surrounding environment. In preparation for the MEG-measurement, each participant’s head shape was digitized using a Polhemus FASTRAK. The participant’s head position and head movement were monitored during MEG recordings using head-position indicator coils. Anatomical MRIs were acquired using hi-res Sagittal T1 weighted 3D IR-SPGR (inversion recovery spoiled gradient echo) images by a GE MR750 3 Tesla scanner with the following pulse sequence parameters: 1 mm isotropic resolution, FoV 240 × 240 mm, acquisition matrix: 240 × 240, 180 slices 1 mm thick, bandwidth per pixel = 347 Hz/pixel, flip angle = 12 degrees, TI = 400 ms, TE = 2.4 ms, TR = 5.5 ms resulting in a TR per slice of 1390 ms.

### Data processing

The acquired MEG data were preprocessed using MaxFilter (-v2.2) (Taulu et al. 2004; Taulu and Simola 2006), and subsequently analyzed and processed using the Fieldtrip toolbox (Oostenveld et al. 2011) in MATLAB (Version 2016a, Mathworks Inc., Natick, MA), as well as the MNE-Python software (Gramfort et al. 2013). Cortical reconstruction and volumetric segmentation of all participants’ MRI was performed with the Freesurfer image analysis suite (Fischl 2012).

#### Preprocessing

MEG data were MaxFiltered by applying temporal signal space separation (tSSS) to suppress artifacts from outside the MEG helmet and to compensate for head movement during recordings (Taulu et al. 2004; Taulu and Simola 2006), before being transformed to a default head position. The tSSS had a buffer length of 10 s and a cut-off correlation coefficient of 0.98. The continuous MEG data were divided into 1 s long epochs from stimulus onset (i.e. onset of each tone). Epochs were then visually inspected for artifacts and outliers with high variance were rejected using *ft_rejectvisual* (Oostenveld et al. 2011). After cleaning, the remaining approximately 70% of all epochs were kept for further analyses.

#### Behavioral data analysis

To assess response accuracy in the MDT task, mean task performance scores for each participant were calculated as the percentage of correct responses out of all 28 responses.

#### Sensor-space analysis of MEG data

Two participants were excluded due to less-than-chance performance in the behavioral task, resulting in 26 participants for all sensor-space MEG analyses. Sensor-space analysis was carried out on cleaned MEG epochs obtained after the preprocessing steps above, to extract the ASSR power for each condition. Firstly, a 30–50 Hz bandpass filter was applied to the epochs which were then averaged per condition, resulting in the *timelocked ASSR*.

For analyses comparing overlapping and nonoverlapping ASSR power, the 200–700 ms (from tone onset) time window was extracted from the *timelocked ASSR* and zero-padded to 1 s before applying a fast Fourier transform (Hanning-tapered, frequency resolution = 1 Hz) to acquire the ASSR power spectra. These analyses ignored the attention conditions and combined data across attend low and attend high conditions, averaging over ~200 cleaned epochs per voice. For each participant, the ASSR power spectra were further averaged across all MEG sensors, before the ASSR power at *f*_m_ (defined as 39 and 43 Hz for the Low and High voices, respectively) was extracted to give the mean ASSR power per voice for the nonoverlapping (ASSR1) and overlapping (ASSR2) experiments.

For analyses involving only the current overlapping experiment, the time window used was 200–700 ms (from tone onset) for the first half of the tone, and 1200–1700 ms (from tone onset) for the second half of the tone. These include the analyses concerning top-down and bottom-up attention. Subsequent steps to extract the ASSR power per voice × bottom-up attention × top-down attention were identical to those described above. The minimum number of cleaned epochs averaged was ~100 per condition. The first second relative to tone onset was considered to experience a bottom-up attention effect as a perceptually distinct change of tone pitch is expected to draw attention towards itself in a bottom-up manner, whereas the second half of the tone was simply a continuation of the same tone and would not have the same salient effect. Hence, in all analyses pertaining to bottom-up attention, we compared the ASSR power during the first 200–700 ms (with bottom-up attention) versus the ASSR power during 1200–1700 ms from tone onset which falls within the second half of the tone (no bottom-up attention). Finally, the mean ASSR power was converted to the base 10 logarithmic scale (lg(power)) to achieve more normal data distributions across participants for parametric statistical analysis (i.e. *t*-tests and ANOVAs).

#### Source-space analysis of MEG data

In addition to the participants excluded due to less-than-chance performance in the behavioral task, a third participant was excluded due to unsuccessful MRI collection, resulting in a final sample of 25 participants for the source-space MEG analyses. We used a minimum-norm estimate (MNE) (Gramfort et al. 2013) distributed source model containing 20,484 dipolar sources on the cortical surface to produce individual-specific anatomical layouts of the ASSR sources. These models were generated by entering sensor-space *timelocked ASSR* data into the MNE computation, before applying a fast Fourier transform (hanning windowed, frequency resolution = 1 Hz), with zero-padding to 1 s. Subsequently, the individual MNE solutions were morphed to a common “fsaverage” template. Cortical maps of vertex-level across-subject *t*-scores were provided for exploratory and visualization purposes only, and not used for hypothesis testing. The rest of this section outlines the additional steps involved in further source-spaces analyses.

#### Difference in ASSR power between overlapping and nonoverlapping experiments

We compared the extent of ASSR power suppression across cortical lobes, due to the inclusion of a second simultaneous voice. For this analysis, we divided the cortical sheet into the left and right frontal, temporal, and parietal lobes according to Brodmann areas designated by the PALS-B12 atlas (Van Essen 2005) (illustrated in Fig. 4). These lobes were selected based on previous results showing that these areas contain most of the strongest ASSR sources (Manting et al. 2020). The mean ASSR power across all vertices within each lobe was computed, and the ASSR power at *f*_m_ was extracted at 39 and 43 Hz for the Low and High voice, respectively. The amount of ASSR power suppression was expressed as a ratio of ASSR2/ASSR1, before taking its lg-value, lg(ASSR1/ASSR2), for the repeated-measures ANOVA.

#### Difference in ASSR source distribution between overlapping and nonoverlapping experiments

To examine how neural resources are distributed to generate ASSR activities in both overlapping and nonoverlapping cases irrespective of their respective total ASSR power, we normalized ASSR1 and ASSR2 by dividing the ASSR power at each vertex over the sum of the ASSR power across all 20,484 vertices at a single-subject level, producing the corresponding SD1 and SD2. This expressed the power of each source (i.e. vertex) as a fraction of the total ASSR power and allowed us to average across voices. We performed a cluster-permutation test (Maris and Oostenveld 2007) to assess whether there exists any region within the frontal, temporal, or parietal lobe that statistically drive the difference in ASSR source distribution between the overlapping and nonoverlapping cases. For this step, a *t*-statistic was computed for every vertex using the difference between SD1 and SD2. Clusters were defined as contiguous vertices across space with *t* > 2.06, and computed using a spatial resolution of 20,484 vertices. The sum of *t*-values within each cluster (*T*_sum_) was computed and compared against a distribution of the largest *T*_sum_ obtained from each of 1,024 random permutations of the conditions (SD1 and SD2) prior to clustering. The difference was defined as significant if there existed any clusters with a *P*-value below 0.05.

#### Top-down and bottom-up attentional enhancement of overlapping-voices ASSR power across lobes and hemispheres

The cortical sheet was divided into 6 lobes, namely left and right frontal, temporal and parietal lobes, according to the PALS-B12 atlas (Van Essen 2005) as described earlier in the section *Difference in ASSR power between overlapping and nonoverlapping experiments*. For each lobe, the mean ASSR2 power across all constituent vertices per voice × attention condition was computed for top-down and bottom-up attention separately. The attentional modulation per lobe was expressed as an AU ratio between the attend and unattend conditions for each voice, and then averaged across the 2 voices. The AU ratios were then compared against an expected mean value of 1 (i.e. when attend = unattend) in a series of one-tailed *t*-tests for positive attentional enhancement at a critical level of 0.05 with Bonferroni correction over six tests. In Table 1, the AU ratios are expressed as a percentage such that an AU of 1.18 corresponds to 18% attentional enhancement.

**Table 1:** ASSR power modulation across cortical lobes by a) top-down and b) bottom-up attention

	Frontal		Temporal		Parietal	
	LH	RH	LH	RH	LH	RH
a) Mean ASSR power modulation by top-down attention	18%	−5%	11%	12%	6%	8%
*P*_one-tailed_ (uncorrected)	0.0050^*^^*^^^^	0.21	0.067	0.028^*^	0.16	0.035^*^
	Frontal		Temporal		Parietal	
	LH	RH	LH	RH	LH	RH
b) Mean ASSR power modulation by bottom-up attention	3%	7%	8%	10%	9%	6%
*P*_one-tailed_ (uncorrected)	0.24	0.13	0.083	0.00072^*^^*^^*^^^^^	0.035^*^	0.026^*^

^*^P < 0.05, ^*^^*^P < 0.01, ^*^^*^^*^P < 0.001 (uncorrected).

^^^ *P* < 0.05, ^^^^*P* < 0.01 (corrected).

## Results

### Behavioral results

Results from the overlapping MDT task showed that 26 out of 28 participants performed significantly above the chance level of 33% (*M* = 70%, SD = 25.1%; *t*(28) = 7.7, *P*_two-tailed_ < 0.001). For the nonoverlapping MDT task, 71% of these 28 participants scored above chance level (SD = 18.6%; *t*(28) = 10.9, *P*_two-tailed_ < 0.001). There is no significant difference in performance scores between the nonoverlapping and overlapping MDT tasks (*P*_two-tailed_ = 0.74).

### MEG results

The results of this work can be summarized from the following perspectives: (3.2.1) comparing the ASSR power and (3.2.2) source distribution during overlapping and nonoverlapping auditory processing, as well as (3.2.3) extracting the modulation effects of top-down and bottom-up auditory attention on the overlapping ASSR.

#### Differences in ASSR power between overlapping and nonoverlapping experiments

**Fig. 2:**
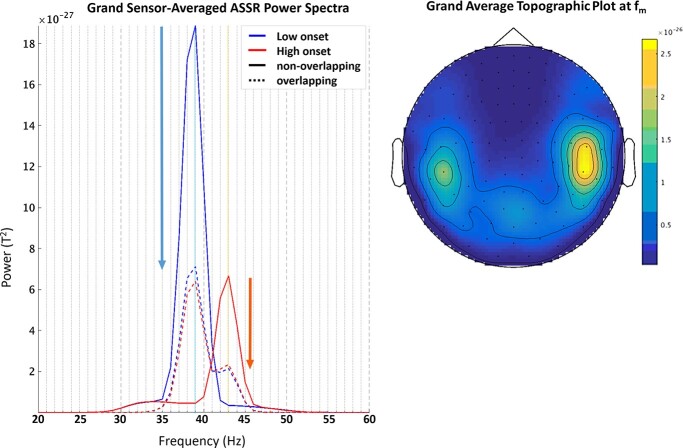
(Left) Grand average nonoverlapping (—) and overlapping (− −) ASSR power spectra during low tone (blue) and high tone (red) onset. Log-transformed ASSR power at *f*_m_ (low: 39 Hz; high: 43 Hz) between nonoverlapping and overlapping conditions was significantly different (*P* < 0.001) when averaged across all MEG sensors. The ASSR decreased to approximately a third of its nonoverlapping power upon the addition of another simultaneous voice as illustrated by downward arrows above. (Right) Grand average topographic plot of MEG gradiometers at *f*_m_ across all 26 subjects. Units are converted to *T*^2^. While the example above depicts the MEG sensor activity pattern for the overlapping High voice at *f*_m_ = 43 Hz, similar patterns of activity were observed for the Low voice at *f*_m_ = 39 Hz, and for the nonoverlapping voices.

Across all MEG sensors, the overlapping ASSR2 is generally lower in power than the nonoverlapping ASSR1. The difference is significant for both the Low voice at *f*_m_ = 39 Hz (*t*(25) = 15.7, *P* < 0.001), and High voice at *f*_m_ = 43 Hz (*t*(25) = 12.2, *P* < 0.001). Figure 2 below shows that the ASSR2 power decreases to approximately a third of the ASSR1 power. The difference in power between the Low and High voice is also significant for both ASSR1 (previously reported (Manting et al. 2021)) and ASSR2 (*t*(25) = 8.9, *P* < 0.001). This is likely due to the lower volume settings of the high compared to the Low voice that elicited a weaker High voice ASSR (Ross et al. 2000).

**Fig. 3:**
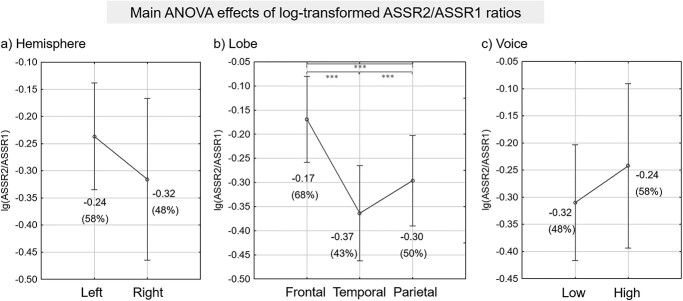
Effect of ASSR power suppression due to simultaneous voices. ANOVA results reported significant main effects of (a) hemisphere (*P* = 0.020), (b) lobe (*P* < 0.001), and (c) voice (*P* = 0.013), but no significant interaction between any of these factors. Post-hoc Tukey tests revealed that all possible combinations of the 3 lobe levels were significantly different (*P* < 0.001, corrected for all combinations as indicated by the asterisks^*^^*^^*^ above), with the temporal lobe experiencing the most suppression from simultaneous sources followed by parietal then frontal. The corresponding ASSR2/ASSR1 ratios, displayed in parentheses, were calculated directly from each respective mean lg(ASSR2/ASSR1) value right above. Vertical bars denote 0.95 confidence intervals.

**Fig. 4:**
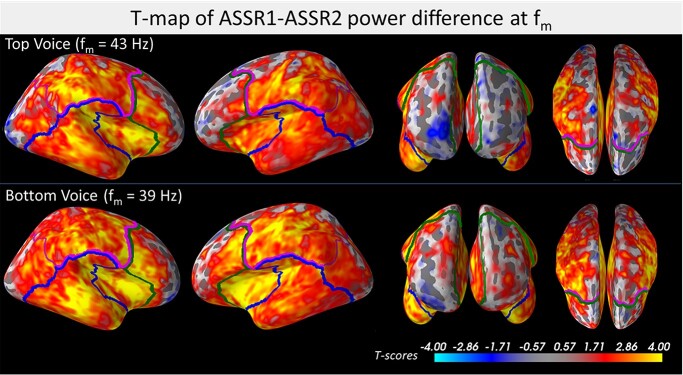
Demarcation of the areas that define each of the frontal (green), temporal (blue), and parietal (magenta) lobes used for ANOVA analysis of the ASSR suppression due to simultaneous voices. For visualization, these demarcations are superimposed over cortical maps of *t*-scores (across-subject) illustrating the power difference between ASSR1 and ASSR2 at vertex-level. Orientation views from left to right: right lateral, left lateral, frontal, top.

Using the source level MNE solutions for ASSR1 and ASSR2, a repeated-measures ANOVA was used to test the differences in this power suppression effect across the bihemispheric frontal, temporal and parietal lobes, with the three factors hemisphere (left/right), lobe (frontal/temporal/parietal) and voice (Low/High). The results showed significant main effects of all 3 factors, hemisphere (*F*[1, 24] = 6.2, *P* = 0.020), lobe (*F*[2, 48] = 69.0, *P* < 0.001) and voice (*F*[1, 24] = 7.3, *P* = 0.013), but no significant interaction between any factor (minimum *P* value = 0.39). Figure 3 plots the mean lg(ASSR2/ASSR1) ratios per level for each of the three factors tested, with ASSR2 expressed as a percentage of ASSR1 (converted from lg-values) displayed below in parenthesis for easier interpretation. Post-hoc Tukey tests of the lobe factor (Fig. 3b) revealed significant differences between all three possible lobe combinations (*P* < 0.001, corrected) with the temporal lobe showing the largest suppression from simultaneous sources (reduced to 43%), followed by parietal (reduced to 50%) then frontal (reduced to 68%). The vertices used for computing the mean ASSR power of each of the three lobes are demarcated in Fig. 4 (frontal: green, temporal: blue, parietal: magenta). For illustration purposes, these lobe demarcations are superimposed over cortical maps of vertex-level across-subject *t*-scores of the power difference between ASSRs generated by nonoverlapping and overlapping voices (ASSR1–ASSR2).

#### ASSR source distribution during the processing of nonoverlapping and overlapping melodies

**Fig. 5:**
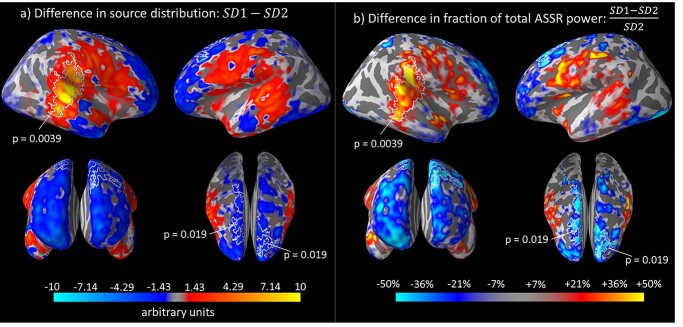
Difference in the distribution of ASSR sources generated by overlapping and nonoverlapping voices across the frontal, temporal, and parietal cortices. Each source (vertex) was normalized by division over the sum of the ASSR power across all 20,484 vertices at a single-subject level, thereby expressing the power of each source as a fraction of the total ASSR power. These values were also averaged across the Low and High voices. The across-subject grand average difference in normalized source power across the cortical space between the nonoverlapping and overlapping experiments (i.e. SD1–SD2) in (a) is shown as a percentage of the normalized overlapping-voices ASSR power in (b) for easier interpretation. All clusters with *P* < 0.05 obtained from a cluster-based permutation test of the nonoverlapping minus overlapping difference (SD1–SD2) are demarcated in white with labeled corresponding *P*-values. For visual clarity, the figure in (b) only includes vertices at least 10 times the median SD1–SD2 power. The results suggest that up to 50% more resources were proportionally allocated from the temporal–parietal regions to the frontal regions when 2 simultaneous voices instead of one were processed. Orientation views (clockwise starting from top-left): right lateral, left lateral, frontal, top.

From a different but related perspective, the inhomogeneity in ASSR suppression (and possible enhancement) can also be observed from the differences in normalized source distribution between the ASSRs generated by nonoverlapping (SD1) and overlapping (SD2) voices. Upon inspection of the spatial differences between SD1 and SD2 (refer to Fig. 5), a larger fraction of total neural resources (+10 to +50%, depending on lateral hemisphere) was activated in temporal–parietal regions during the nonoverlapping compared to overlapping experiment. In contrast, frontal regions received a lower proportion of allocated neural resources (−20 to −50%) when listening to nonoverlapping compared to overlapping voices. A cluster permutation test yielded significant differences between SD1 and SD2. This result was driven by clusters in the right temporal cortex (*P* = 0.0039) and bi-hemispheric fronto-parietal areas (*P* = 0.019) which are marked in white in Fig. 5. Taken together with the above ASSR power difference results, these results showed variations in the degree of suppression across cortical regions owing to the additional concurrent ASSR source.

#### ASSR modulation from top-down and bottom-up attention during the overlapping experiment

**Fig. 6:**
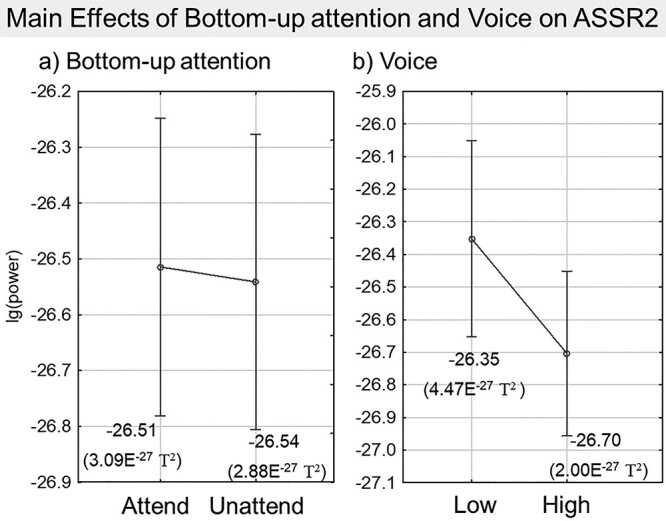
Effect of bottom-up attention and voice on overlapping-voices ASSR2 power. ANOVA results reported significant main effects of (a) bottom-up attention (*P* = 0.0011) and (b) voice (*P* < 0.001), but no significant interaction between them (*P* = 0.37). The mean lg(power) values are plotted with their equivalents in *T*^2^ scale displayed in parentheses below. Vertical bars denote 0.95 confidence intervals.

**Fig. 7:**
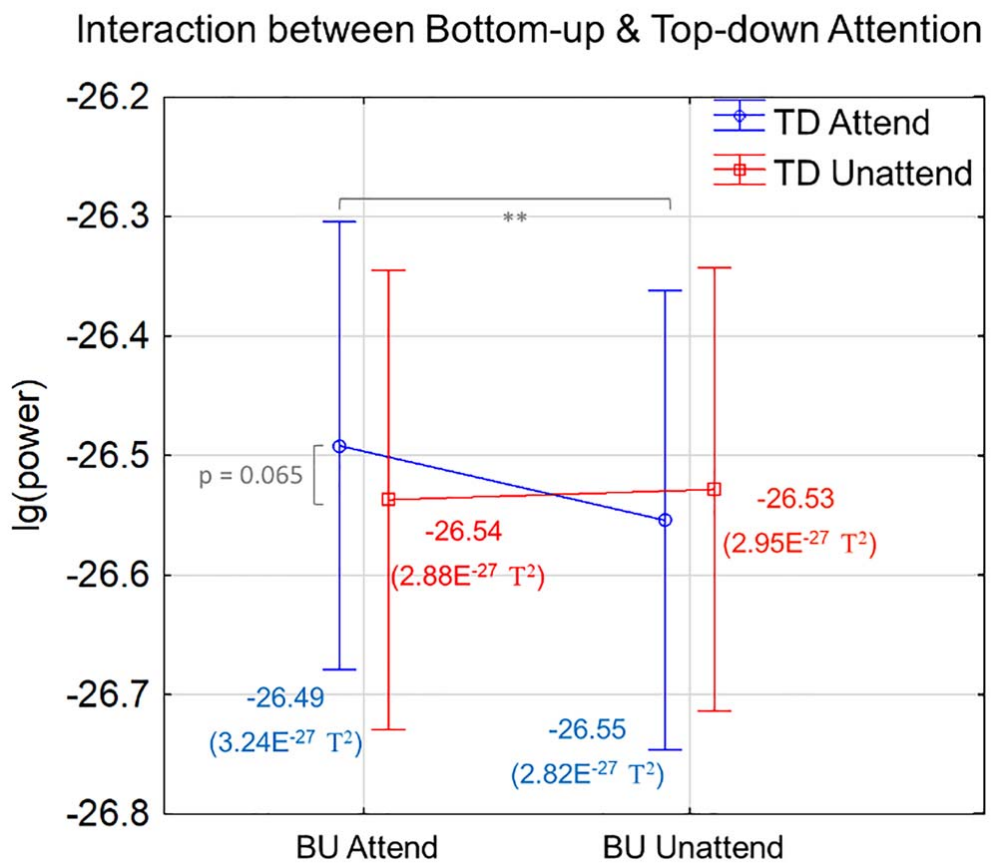
Interaction between bottom-up (BU) and top-down (TD) attentional modulation of ASSR2 power. A 3-factor ANOVA between TD attention, BU attention and voice revealed significant interaction between TD and BU attention (*P =* 0.0072). Post-hoc Tukey tests of this interaction revealed that the only significant difference (*P* = 0.0067) was between the BU attend (left column) and BU unattend (right column), during the TD attend condition. The difference between TD attend and TD unattend when BU attention engaged (BU attend) missed significance when using a 2-tailed test (*P* = 0.065), although it would have been significant if a 1-tailed test was used instead to test only for the effect of attentional enhancement. The mean lg(power) values are plotted with their equivalents in *T*^2^ scale displayed in parentheses below. Vertical bars denote 0.95 confidence intervals.

Using data averaged across all MEG sensors, a repeated-measures ANOVA tested the modulation in ASSR2 power by top-down driven attention (TD attend/unattend) × bottom-up driven attention (BU attend/unattend) × voice (Low/High). There was a significant main effect of bottom-up attention (*F*[1, 25] = 7.5, *P =* 0.0011) and voice (*F*[1, 25] = 74.0, *P <* 0.001) but not top-down attention (*F*[1, 25] = 0.8, *P =* 0.39). Figure 6 plots the mean lg(power) per level for the significant main effects of bottom-up attention and voice, with lg-values converted to *T*^2^ scale displayed in parentheses. Only the interaction between top-down attention and bottom-up attention was significant (*F*[1, 25] = 8.5, *P =* 0.0072). Post-hoc Tukey tests of this interaction revealed that the only significant difference was between the BU attend and BU unattend when top-down attention was active (15% bottom-up attentional enhancement; *P* = 0.0067). The difference between TD attend and TD unattend when bottom-up attention was strongly engaged during tone onset was close to significance (10% top-down attentional enhancement; *P* = 0.065). As the Tukey test computes the two-tailed *P*-value by default, a *P*-value of 0.065 would have theoretically passed significance if a one-tailed *P*-value was used instead, which in this case we have valid grounds to test only the unidirectional enhancement effect of attention. As expected, the ASSR power of the attended tone is strongest during the tone onset, when both bottom-up and top-down attention are in play. Figure 7 summarizes these interaction results, displaying lg(power) values and their *T*^2^ equivalents in parentheses. Additionally, there was no significant interaction between voice and top-down (*F*[1, 25] = 0.6, *P* = 0.43) or bottom-up attention (*F*[1, 25] = 0.8, *P* = 0.37), suggesting that voice does not affect the attentional modulation (as explained earlier, the effect of voice is likely attributed to the lower volume settings of the high compared to the Low voice that elicited a weaker High voice ASSR (Ross et al. 2000)).

Source analyses of the top-down attentional modulation across lobes and hemispheres yielded significant ASSR2 power enhancement by top-down selective attention at the left frontal cortical lobe (*M*_AU_ = 1.18, SD = 0.32; *t*(25) = 2.80, *P*_one-tailed, corrected_ = 0.03). Similarly, we carried out source-level analyses to find out if the bottom-up attentional modulation is significant across lobes and hemispheres. *T*-tests results revealed significant ASSR2 power enhancement by bottom-up selective attention at the right temporal cortical lobe (*M*_AU_ = 1.10, SD = 0.14; *t*(25) = 3.60, *P*_one-tailed, corrected_ = 0.0043). For reference, the complete result containing the respective uncorrected *P*-value and mean top-down and bottom-up attentional modulation for all 6 lobes tested is shown in Table 1a and b, respectively. Lobe demarcations are identical to that depicted in Fig. 4.

## Discussion

In this study, we aimed to characterize the differences in the ~40 Hz cortical ASSR during auditory processing of nonoverlapping versus overlapping voices, comparing ASSR power, ASSR source distribution, and modulation effects on ASSR power from top-down and bottom-up attention. In pursuit of a method to separate mixed neural signals generated by multiple simultaneous sounds into the individual constituent activities, we turn to frequency-tagging as a means to label, identify, and isolate neural ASSRs uniquely, granting us the opportunity to study stimulus-specific neural responses even amidst the presence of other ongoing brain activity. While this approach may appear straightforward in theory, it has proven to be a tricky task to put into practice, owing to the sensitivity of the ASSR to a myriad of factors such as the signal-to-noise ratio, type of stimulus, modulation depth, complexity of task, as well as number of simultaneous auditory sources (John et al. 1998; Ross et al. 2000; Picton et al. 2003; Korczak et al. 2012). To better understand the nature of the ASSR for optimizing its usage in cognitive research, the current study aims to examine the differences in the neural manifestation of the ASSR during the presentation of overlapping versus nonoverlapping frequency-tagged sounds, and whether these differences hamper the ability of the frequency-tagging method to result in clearly resolved neural representations of each source (i.e. if increasing the number of simultaneous sounds would reduce the fidelity of the individual ASSRs). The conclusions related to this objective are discussed in the section *Changes in ASSR power and distribution due to the presence of a simultaneous competing voice*.

Furthermore, we were also interested in examining whether the ASSR’s sensitivity to attentional modulation, as observed in the nonoverlapping case (Manting et al. 2020, 2021), would be retained in the presence of overlapping voices. Hence, we used a cognitively demanding “Melody Development Tracking” task that entailed participants to selectively focus attention to a designated target melody presented within a mixture of competing melodies, and subsequently report the last direction of pitch change in the designated melody when the playback unexpectedly stopped. This paradigm has allowed us to test the feasibility of using the ASSR to study auditory top-down (discussed in the section *Top-down and Bottom-up modulation of ASSRs generated by overlapping-voices*) and bottom-up attention (section *Conclusions, limitations and recommendations for future work*) in complex multivoice auditory scenarios. Ultimately, our goal is to investigate the neural correlates of cognitive processes and their cortical distribution in complex and naturalistic soundscapes, by using frequency-tagging for precise tracking of individual auditory sources. The following section discusses the key insights acquired from our findings in greater detail, in relation to these goals.

### Changes in ASSR power and distribution due to the presence of a simultaneous competing voice

In line with our first hypothesis, we have found that the ASSR decreases to approximately a third of its power when there were 2 overlapping voices compared to nonoverlapping voices. The sum of the Low and High voice ASSR powers in the overlapping case was lower than that in the nonoverlapping case, even though their volume had been equally calibrated to be 75 dB SPL. This is likely due to the nonlinearity of the basilar membrane hair cell transduction process that produces a compressive rectification effect, which causes energy to be distributed to additional frequencies equal to the sums and differences of the modulation and carrier frequencies present simultaneously (Lins and Picton 1995; John et al. 1998). ANOVA analysis using source-space data showed significant hemisphere, lobe and voice differences in the extent of ASSR power suppression due to simultaneous voices. Since ASSR power differences between the Low and High voices (in our case owing to volume differences set to maintain an equal perception of loudness between voices) can potentially confound the results with respect to the voice factor, we omitted the interpretation of the suppression differences across voices. Posthoc analysis revealed that the temporal lobe experienced the largest suppression (reduced to 43%) from simultaneous voices followed by parietal (reduced to 50%) then frontal (reduced to 68%) lobes. When compensating for the fact that ASSR sources from nonoverlapping voices are overall stronger than that of overlapping voices (i.e. by comparing their respective normalized distributions), results showed that the fraction of activated neural resources decreased in the temporal–parietal regions but increased in the frontal regions when listening to overlapping voices.

To bring together the 2 main findings discussed, while the raw ASSR power across all three cortical lobes decreased from using nonoverlapping to overlapping voices, the proportion of total ASSR power per region decreased in the temporal–parietal lobes but increased in the frontal lobe, thus explaining why the frontal raw ASSR power experienced the least degree of suppression from simultaneous ASSRs (because part of this suppression was compensated by the increase in fractional resources allocated to the frontal region). We believe that identifying, separating, selectively directing and maintaining attention to the target voice was more cognitively demanding in the overlapping scenario, leading to more resources being allocated to the frontal region, an area known to be central in the execution and maintenance of selective attention (Foster et al. 1994; Cohen 2014; Plakke and Romanski 2014). To the best of our knowledge, this is the first time any study has attempted to spatially characterize the effect of an additional overlapping AM sound on the ASSR power and its source distribution, thus advancing our understanding of the ASSR and its potential applications.

### Top-down and bottom-up modulation of ASSRs generated by overlapping-voices

At sensor level, our ANOVA results showed that bottom-up selective attention to one voice increases its corresponding ASSR power by approximately 15% in the overlapping setup. On the contrary, the effect of top-down selective attention on the overlapping-voices ASSR power failed to reach significance, showing that the additional overlapping voice impeded the ability of the ASSR to pick up the attentional enhancement This agrees with our initial hypothesis which predicted that compared to nonoverlapping voices, overlapping-voices would generate ASSRs that are less sensitive to modulation from top-down selective attention, possibly due to weaker signal-to-noise ratios owing to the suppression effect from simultaneous voices.

At source level, several cortical lobes showed significant overlapping-voices ASSR enhancement by top-down attention ranging from 8 to 18%, although the enhancement was lower than previous results (Manting et al. 2021) of 13–30% for ASSRs in the nonoverlapping case. These findings reinforce the “gain” theory of selective attention (Hillyard et al. 1973, 1998; Mesgarani and Chang 2012; Ding and Simon 2012a, 2012b), wherein attention enhances the neural representation of a stimulus. In the overlapping-voices experiment, top-down attentional enhancement appeared to manifest most apparently in the frontal lobe, as predicted by our hypothesis. This finding is consistent with the notion of a frontal-based center of attentional control championed by numerous literature (Foster et al. 1994; Pugh et al. 1996; Tzourio et al. 1997; Cohen 2014; Plakke and Romanski 2014). On the other hand, the effect of bottom-up attention was most significant in the temporal lobe, where the lower level sensory auditory cortices are situated. Again, this result confirms our hypothesis stating that bottom-up attention will be centered at the temporal regions. One possible explanation to these findings could be that involuntary bottom-up attention is associated with automatic stimulus processing mechanisms that predominate in the sensory cortices (Huang et al. 2012), in contrast to how top-down factors such as greater utilization of voluntary attention and working memory modulate higher-level executive regions located frontally (Foster et al. 1994; Huang et al. 2012; Cohen 2014; Plakke and Romanski 2014). This explanation is coherent with studies demonstrating that top-down and bottom-up mechanisms are mediated by specialized neural networks, albeit with partially overlapping regions (Salmi et al. 2009; Huang et al. 2012). Thus, the above findings build on our previous work (Manting et al. 2020; Manting et al. 2021) that demonstrated the feasibility of using the ASSR to extract attentional effects by extending the application from voices separated in time to overlapping voices. A key aspect of this study that stands out from the existing literature is that it draws upon sound separation based on perceptual features (in this case, pitch and timing) rather than location (i.e. left-versus-right ear) as in the majority of related work in this field (Müller et al. 2009; Lazzouni et al. 2010; Bharadwaj et al. 2014). This is important and relevant as feature-based sound separation and identification is a significant component of selective attention in natural cocktail party-like settings.

Moreover, additional analysis into interaction effects between bottom-up and top-down attention revealed a synergistic relationship between the 2, in that the enhancing effect of top-down attention was more observable when bottom-up attention was also directed towards the same tone and vice versa. This is in-line with the findings of Shuai and Elhilali (2014) who found out that bottom-up driven increases in ASSR due to salient events supplemented ASSR increases due to top-down attention. Perceptually, we postulate that bottom-up driven attention based on stimulus saliency on tone onset may help the listener to “find” the tone more quickly and consciously direct resources towards it via top-down attentional mechanisms. However, top-down direction of attention towards a target tone can also be counteracted by bottom-up attention towards a competing simultaneous tone, which may explain why the effect of top-down attention on the cued voice was not observable when the other competing voice experienced a salient change in pitch (and thus drew attention away from the cued voice through bottom-up mechanisms). This agrees with Huang and Elhilali’s recent study (Huang and Elhilali 2020) showing how salient distractors in the background suppress neural responses to the attended sequence that counters top-down enhancement effects. Furthermore, the authors showed that the amount of suppression increases with higher degree of distractor saliency. By similar arguments, top-down attention towards a target voice suppresses attentional resources towards the rival voice, explaining why no effect of bottom-up attention was observed in the unattended voice. Consistent with earlier studies (Shuai and Elhilali 2014; Bidet-Caulet et al. 2015; Huang and Elhilali 2020), our current results support the idea that bottom-up and top-down attentional mechanisms dynamically coordinate and compete in the human brain. As studies that directly address the effect of bottom-up attention on the ASSR are rare, even more so for how the interplay between top-down and bottom-up attention affects the ASSRs to both the attended and competing stream, the present work offers refreshing and novel insights into the field.

### Conclusions, limitations, and recommendations for future work

To summarize, we discovered that the suppression of ASSR power due to an additional simultaneously overlapping voice was not homogeneous across the neural cortex, with more suppression in the temporal–parietal regions than in the frontal regions. This was partly attributed to the reallocation of total neural resources from temporal–parietal to frontal areas during the processing of overlapping voices, which can be explained by the notion that the higher complexity in the overlapping auditory mix engaged more strongly higher-level cognitive processing mechanisms housed in the frontal regions (Friederici 2012; Nourski 2017; O’Sullivan et al. 2019). Furthermore, we learned that while the suppression makes it more difficult for the effect of top-down attention to achieve significance at a general sensor level, significant effects of top-down attentional modulation could still be obtained using a more localized source-level (lobe-level) approach. In relation, the effect of ASSR enhancement due to bottom-up auditory attention can be observed both at sensor and source level. While the effects of top-down and bottom-up attention seemed to complement one another in terms of power enhancement, they were concentrated at different regions, with top-down attention strongest at the frontal lobe and bottom-up attention was centered at the lower-level auditory sensory areas.

While our current approach provides statistical evidence demonstrating the attentional enhancement of the ASSR at lobe-level, this method includes many vertices with low signal power and weak attentional modulation, thus diluting and reducing the average extent of effect compared to our earlier studies (Manting et al. 2020, 2021) that used regions-of-interests with fewer, strongly activated source vertices. Possibly as a result, the top-down attentional enhancement in areas that showed evident enhancement previously, namely the temporal and parietal lobes, missed significance in the current study (although they were significant before corrections for multiple comparisons). One major limitation that precludes our present analysis from demarcating smaller regions-of-interests is the presence of spatial variation between individuals in the attentional modulation, especially at the vertex level. Hence, alternative methods to characterize the area of effect while accounting for such individual variation are needed for more precise mapping of the ASSR attentional modulation. Moreover, a more concentrated approach with higher signal power opens up more methods for analyzing the ASSR, for example, to study its attentional modulation across time.

Additionally, the present findings also provide evidence supporting the interpretation that the ASSR sources in the frontal and temporal lobes are independent, characteristically different, as well as functionally distinct. Firstly, these sources were suppressed to different degrees when a competing simultaneous voice was added. In relation, the frontal and temporal sources also varied in their proportional strength of activation during single- versus simultaneous-voice listening. Thirdly, the amount of modulation by top-down and bottom-up attention was different between these sources. Furthermore, our previous work (Manting et al. 2021) has also shown that the activation patterns of the frontal and temporal ASSR sources vary distinctly across time, particularly during and after stimulus playback.

In conclusion, the aforementioned novel findings of the present work advance the application of frequency-tagging and ASSR research, and contribute a significant leap towards separating and extracting neural signals in complex soundscapes, from the conventional simplistic experimental set-ups. Eventually, this would pave the way towards applying frequency-tagging to more naturalistic soundscapes, with greater degree of complexity, for translating experimental findings into explanations of real-life cognitive phenomena.
